# Low-cost detection of norovirus using paper-based cell-free systems and synbody-based viral enrichment

**DOI:** 10.1093/synbio/ysy018

**Published:** 2018-09-19

**Authors:** Duo Ma, Luhui Shen, Kaiyue Wu, Chris W Diehnelt, Alexander A Green

**Affiliations:** 1 Biodesign Center for Molecular Design and Biomimetics, The Biodesign Institute and School of Molecular Sciences, Arizona State University, AZ, USA; 2 Biodesign Center for Innovations in Medicine, The Biodesign Institute and School of Molecular Sciences, Arizona State University, AZ, USA

**Keywords:** diagnostic, riboregulator, norovirus, cell-free system

## Abstract

Noroviruses are a primary cause of gastroenteritis and foodborne illness with cases that affect millions of people worldwide each year. Inexpensive tests for norovirus that do not require sophisticated laboratory equipment are important tools for ensuring that patients receive timely treatment and for containing outbreaks. Herein, we demonstrate a low-cost colorimetric assay that detects norovirus from clinical samples by combining paper-based cell-free transcription–translation systems, isothermal amplification and virus enrichment by synbodies. Using isothermal amplification and cell-free RNA sensing with toehold switches, we demonstrate that the assay enables detection of norovirus GII.4 Sydney from stool down to concentrations of 270 aM in reactions that can be directly read by eye. Furthermore, norovirus-binding synbodies and magnetic beads are used to concentrate the virus and provide a 1000-fold increase in assay sensitivity extending its detection limit to 270 zM. These results demonstrate the utility of paper-based cell-free diagnostic systems for identification of foodborne pathogens and provide a versatile diagnostic assay that can be applied to the concentration, amplification and detection of a broad range of infectious agents.

## 1. Introduction

Noroviruses are the leading cause of human gastroenteritis^1^ and globally are estimated to exact $60 billion in societal costs each year.^2^ These viruses are extremely contagious^3^ and can persist in the environment on contaminated surfaces, causing frequent outbreaks in closed settings such as long-term health care facilities, hospitals, schools and cruise ships.^1^ Noroviruses are also the primary cause of foodborne illness,^4^ and thus impose substantial risks to the food industry. Although norovirus infections are often self-limiting with symptoms that persist for only 48 h in healthy individuals, the virus can lead to severe symptoms and prolonged illnesses in young children and the elderly.^1^ In the USA alone, norovirus infections are estimated to impose an annual burden of 400 000 emergency department visits, 1 million pediatric medical care visits and 19–21 million total illnesses.^5^^,^^6^ In developing countries, the virus is associated with poorer health outcomes and is estimated to cause over 200 000 deaths annually.^7^^,^^8^

Methods to accurately diagnose norovirus infections are thus essential to contain the spread of outbreaks and to ensure that patients receive optimal treatment. Noroviruses are non-enveloped, single-stranded, positive-sense RNA viruses and they exhibit substantial genetic variability, which can complicate efforts to develop effective diagnostics. Over 30 norovirus genotypes are known to cause acute gastroenteritis in humans. Of those genotypes, the GII.4 virus is the most commonly reported,^9^^,^^10^ and newly evolved strains in this genotype have emerged to cause global pandemics roughly every 2–3 years since the mid-1990s.^1^^,^^3^ At present, the most commonly used methods to detect norovirus are immunochromatographic lateral flow assays and polymerase chain reaction (PCR)-based tests.^1^ Immunochromatographic lateral flow assays employ antibodies that recognize viral surface proteins and can be advantageous since they do not require specialized equipment and provide test results in 15 min. However, these tests provide limited sensitivity^11^^,^^12^ and their results can be strongly genotype dependent.^1^ Real-time quantitative reverse transcriptase PCR (qRT-PCR) is currently the gold standard for detection of norovirus.^1^ qRT-PCR assays can be targeted to conserved regions of the norovirus genome and they provide high specificity and sensitivity. These assays, however, require expensive thermal cycling equipment and are typically run in centralized laboratories.^13^ Shipment of samples can delay test results and specialized laboratory equipment is often not available in developing countries or in remote settings, such as ships at sea, where outbreaks frequently occur. Highly automated commercial instruments such as the Cepheid GeneXpert have been developed for decentralized use.^13^ However, these instruments are expensive. Even with negotiated prices for low- and middle-income countries, the GeneXpert instrument, for instance, costs $17 000 and has cartridges available at a concessional price of $9.98. These factors lead to an overall per test cost of $14.93 once labor, consumable and other costs are included.^14^ In the absence of discounts, GeneXpert costs rise substantially to $30.26–$155.44 per test depending on the country.^15^

In response to these limitations, researchers have implemented nucleic acid tests for norovirus that employ isothermal amplification methods rather than conventional PCR^16–26^ to obviate the need for expensive equipment and facilitate decentralized assays. Use of isothermal amplification methods, such as nucleic acid sequence-based amplification (NASBA),^27^ loop-mediated isothermal amplification^28^ and recombinase polymerase amplification (RPA),^29^ avoids the need for expensive thermal cyclers, and it has enabled noroviruses to be detected in fecal samples,^16–18^^,^^21–24^^,^^26^ surface water,^19^ and in oysters.^20^^,^^25^ However, these tests have required additional equipment to read out assay results via fluorescence or electrochemiluminescence,^16^^,^^18^^,^^19^^,^^22^^,^^24^^,^^26^ have the potential for false positives if the isothermal amplification is non-specific,^17^^,^^20^^,^^21^^,^^23^^,^^25^ or rely on expensive reagents such as non-canonical or fluorescent DNA bases.^18^^,^^19^^,^^22^^,^^24^^,^^26^ We have recently developed an alternative strategy for nucleic acid detection that employs cell-free transcription-translation reactions embedded on paper substrates.^30^ These systems employ freeze-drying to preserve the activity of the cell-free systems for over a year at room temperature, and they can be reactivated using water to enable synthetic biology tools to be deployed outside the lab. In previous work, these paper-based systems successfully detected the Zika virus when coupled with isothermal NASBA reactions^31^ and employed programmable riboregulators called toehold switches^32^ to directly verify the sequence of the amplified RNA and produce the reporter enzyme ß-galactosidase (lacZ). This Zika assay was low cost at $1 to read out each test, required only inexpensive equipment and provided results that could be detected directly by eye through cleavage of a chromogenic lacZ substrate.

**Figure 1.**
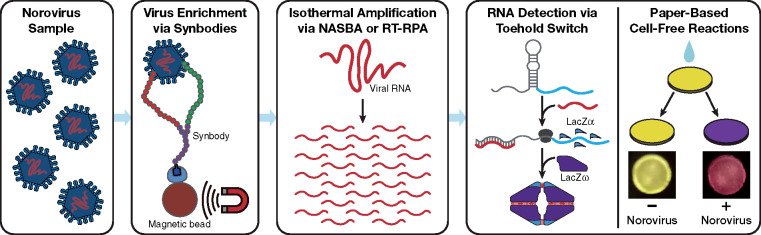
Overview of the norovirus detection assay using paper-based cell-free transcription–translation reactions. A norovirus sample is first enriched using synbodies and viral RNA amplified isothermally using nucleic acid sequence-based amplification (NASBA) or reverse transcriptase recombinase polymerase amplification (RT-RPA). The amplified nucleic acids are added to paper-based cell-free reactions where norovirus RNAs are detected by sequence-specific toehold switches. The toehold switches generate the lacZɑ peptide, which produces a purple-colored product after complementation with lacZω. Samples positive for norovirus can be identified by their purple color following the assay.

Here, we report the application of the paper-based cell-free platform for the detection of the prevalent GII.4 Sydney norovirus genotype (Figure 1). Beginning from fecal samples or a dilute solution containing the virus, the assay employs biotin-labelled synthetic peptide affinity ligands known as synbodies to capture norovirus particles and concentrate them using streptavidin-coated magnetic beads. A brief heating step is used to release the norovirus RNA, and either NASBA or reverse transcriptase RPA (RT-RPA) is employed to amplify the viral RNA. The amplification products are then added to paper-based cell-free reactions where norovirus-specific toehold switches are used to verify their sequences and produce the lacZɑ peptide, which provides a visual reaction readout. We demonstrate that this assay enables detection of norovirus GII.4 Sydney from a stool sample down to concentrations of 270 aM without the use of a concentration step and further show that synbody-based enrichment of the virus can lower the detection limit by 1000-fold to 270 zM. We also demonstrate that the use of ɑ-complementation, in which the lacZɑ and lacZω peptides complement to form the active lacZ enzyme, can reduce the time to detection of the paper-based assay by up to 23 min or 41% compared to experiments employing the full-length lacZ as the toehold switch output. These results expand the range of sample types and viruses that can be analyzed using paper-based cell-free systems and provide new strategies to improve the sensitivity and reduce the time of these inexpensive diagnostic assays.

## 2. Materials and methods

### 2.1 Norovirus samples and bacterial strains

Stool samples positive for the norovirus GII.4 Sydney genotype and the norovirus GI.2 genotype were generously provided by Jan Vinjé from the National Calicivirus Laboratory at the Centers for Disease Control and Prevention (CDC). *Escherichia coli* MG1655 (ATCC, 700926), methicillin-resistant *Staphylococcus aureus* MRSA252 (ATCC, BAA-1720) and *Bacillus subtilis* 168 (ATCC, 23857) were used for assay cross-reactivity experiments. For these experiments, RNA from the bacteria was extracted using a Quick-RNA Fungal/Bacterial Miniprep Kit (Zymo Research) following the manufacturer’s instructions. To obtain purified viral RNA for cross-reactivity experiments, 5 µl of GII.4, GI.2 and GI.6 positive stool samples were suspended in 140 µl RNase-free water. The viral RNA was extracted by using QIAamp DSP Viral RNA Mini Kit (Qiagen, USA) according to the manufacturer’s instructions. RNAs were eluted with 50 µl RNase-free water and stored at −80°C. *Escherichiacoli* DH5ɑ (ThermoFisher Scientific) was used for cloning of toehold switch plasmids.

### *2.2 In silico* selection of toehold switch designs

An updated version of the selection algorithm described previously^32^ was used to identify toehold switches for detection of norovirus RNA. The algorithm facilitated selection six promising designs from a set of over 100 candidate toehold switches generated from each norovirus target RNA. Candidate devices were designed to bind to a 36-nt continuous region of the norovirus target RNA. Putative toehold switches were generated at 1-nt increments along the norovirus target RNA and multiple ensemble defect levels were computed for each sensor based on its deviation from the ideal secondary structure of the toehold switch. Ensemble defects were calculated for the toehold switch 5′ end through to the 3′ end of the hairpin (*d*_min_sensor_), the toehold domain of the toehold switch (*d*_toehold_), the binding site of the toehold switch within the target RNA (*d*_binding_site_) and the toehold switch region starting with the base immediately 3′ of the target RNA binding site and extending 31 nts beyond the last base on the 3′ end of the hairpin (*d*_active_sensor_) (see Supplementary Information and Supplementary Figure S1 for additional details on defect calculations). The parameter *d*_active_sensor_ was intended to provide a measure of any secondary structures in the activated toehold switch that could interfere with translation after binding to the target RNA.

In addition to ensemble defects, the equilibrium fraction *f* of target/toehold switch complexes in a system with equimolar concentrations of target and toehold switch RNAs was calculated as a measure of the affinity of the two RNAs. In practice, this parameter was almost always equal to 1. Designs that produced in-frame stop codons in the output gene were eliminated from further consideration. Each of the parameters was then normalized such that their maximum value across the set of putative designs for a given target RNA was equal to 1. These normalized parameters, designated by an overscore, were then inserted into a scoring function *s*:

$$s=5{\bar{d}}_{toehold}+4{\bar{d}}_{activesensor}+2{\overset{¯-}{d}}_{minsensor\;}+2{\bar{d}}_{bindingsite}+(1-f)$$

Toehold switches displaying the lowest values of *s* and screened to have *f *>* *0.9 were selected for experimental testing. Sequences of the toehold switches generated by the algorithm are provided in Supplementary Table S1 along with those of the norovirus target regions. The weighting coefficients used in the scoring function were determined empirically based on testing of earlier toehold switch mRNA sensor designs.^30^^,^^32^ Two alternative scoring functions informed by experimental data from the norovirus toehold switches are presented in the Supplementary Information and Supplementary Figure S2.

### 2.3 Toehold switch plasmid construction

The list of plasmids used in this work are provided in Supplementary Table S2. Plasmids and DNA templates for transcription were constructed using conventional molecular biology techniques. Synthetic DNA (Integrated DNA Technologies) encoding the norovirus-specific toehold switch sensors was amplified by PCR and inserted into plasmids using Gibson assembly^33^ with 30-bp overlap regions as described previously.^34^ The sequences of the plasmids were confirmed using Sanger sequencing (DNASU Sequencing Core, Tempe, AZ, USA). Sequences of the primers used for plasmid construction are listed Supplementary Table S3. This table lists the source template amplified by each primer pair and indicates what plasmid was produced following Gibson assembly of the resulting PCR products. The synthetic DNA sequences used to generate toehold switches for insertion into plasmids are listed in Supplementary Table S4. This table also contains the primers used for Sanger sequencing of the plasmids.

### 2.4 Preparation of paper-based cell-free systems

Cell-free transcription–translation systems (NEB, PURExpress) were prepared for freeze-drying with the following components by volume: cell-free solution A, 40%; cell-free solution B, 30%; RNase Inhibitor (Roche, 03335402001, distributed by MilliporeSigma), 2%; chlorophenol red-b-D-galactopyranoside (Roche, 10884308001, distributed by MilliporeSigma, 24 mg/ml), 2.5%; with the remaining volume reserved for toehold switch DNA, water and lacZω peptide added to a final concentration of 2 µM. When testing the toehold switches expressed from a plasmid, the plasmid DNA was added to the cell-free reaction mix to a final concentration of 30 ng/µl. When testing toehold switches expressed from linear DNA, the DNA was added to the cell-free reaction mix to a final concentration of 33 nM.

Filter paper (Whatman, 1442-042) for housing the cell-free reactions was first blocked with 5% bovine serum albumin (BSA) overnight. After blocking, the paper was washed three times in water for 5 to 10 min. The paper was then heated to 50°C for drying and cut into 2-mm diameter paper disks using a biopsy punch. The disks were transferred into 200-µl PCR tubes and 1.8 µl of the cell-free reaction mix was applied to each disk. PCR tubes containing the paper disks were then flash frozen in liquid nitrogen and transferred into a lyophilizer to dry overnight. Measurements were performed on the resulting paper disks 2–4 days after the freeze-drying process was completed. The paper disks remained active for at least a month of room temperature storage using conditions described previously,^30^ with the systems stored under nitrogen, shielded from light and in the presence of silica gel desiccation packages.

### 2.5 Screening of norovirus-specific toehold switches

Norovirus target RNA was produced using T7 RNA polymerase-based transcription (Epicenter, ASF3257) from linearized DNA templates. 1.8 µl of a 5 µM solution of the target RNA was applied to a paper disk containing the embedded cell-free system and DNA for the toehold switch. The progress of the cell-free reaction was then monitored in a plate reader (Biotek, H1MF) at 37°C in triplicate. The relative absorbance of the paper-based reactions at 575 nm wavelength or OD575 was calculated by taking the absorbance at 575 nm and subtracting from it the absorbance at 575 nm measured at the start of the reaction. This relative absorbance thus removes any absorbance contribution from the paper disk and the lacZ substrate chlorophenol red-b-D-galactopyranoside. The fold change in lacZ production rate was calculated by computing the rate of change in OD575 and dividing the rate obtained for the toehold switch in the presence of the target RNA by that obtained in the absence of the target RNA. The fold change in lacZ production rate was measured after 1 h of cell-free reaction for assessment of the toehold switches. The change in OD575 or ΔOD575 was calculated by taking the OD575 for the reaction with the toehold switch and the target RNA and subtracting from it the OD575 for the reaction of the toehold switch without the target RNA. ΔOD575 was computed after 2 h of cell-free reaction. Errors in OD575 were determined from the standard deviation of triplicate measurements. Errors in fold change lacZ production rate and ΔOD575 were determined by adding the relative and absolute errors of OD575 in quadrature, respectively. Welch’s unequal variances t-test was used to calculate *P*-values for plate reader detection experiments with *P* < 0.05 used as the cutoff to define a statistically significant result.

### 2.6 Isothermal amplification of norovirus RNA

For NASBA experiments, reaction buffer (Life Sciences, NECB-24; 33.5%), nucleotide mix (Life Sciences NECN-24; 16.5%), RNase inhibitor (Roche, 03335402001; 0.5%), 12.5 mM of each DNA primer (2%), nuclease free water (2.5%) and RNA amplicon (20%) were assembled at 4°C and incubated at 65°C for 2 min, followed by a 10-min incubation at 41°C. Enzyme Mix (Life Sciences NEC-1-24; 25%) was then added to the reaction (for a final volume of 5 µl), and the mixture was incubated at 41°C for 2 h. The amplified product was then diluted 1:6 in water and applied to paper disks containing the cell-free system and DNA for the toehold switch. Sequences of the primers used for NASBA and RT-RPA are provided in Supplementary Table S5.

RT-RPA experiments used the commercial TwistAmp Basic RT kit (TwistDx). Reactions were prepared by combining 10 µM forward primer (4.8%), 10 µM reverse primer (4.8%), rehydration buffer, RNase Inhibitor (Roche, 03335402001; 4.4%) and RNA amplicon (22%) at room temperature and transferring the mixture to the freeze-dried reaction pellet. After mixing, 2.5 µl of 280 mM magnesium acetate (5%) was added to start the reaction, and it was incubated at 41°C for 5–7 min. The reaction tube was then inverted vigorously 8–10 times, spun down briefly, and returned to incubation at 41°C for 2 h. The amplified product was then diluted 1:6 in water and applied to paper disks containing the cell-free system and DNA for the toehold switch.

For determination of assay detection limits, NASBA and RPA reactions were run in triplicate for each concentration of the target RNA or virus and applied to the paper-based toehold switch reactions as described above.

### 2.7 Synbody-based virus enrichment

A 30-µl volume of MyOne Streptavidin C1 streptavidin-coated magnetic beads (Life Technologies, USA), corresponding to 2.1 × 10^8^ to 3.6 × 10^8^ total beads, was added to Protein LowBind tubes (Eppendorf, USA). The bead storage solution was removed and the beads were washed three times with 1 ml of PBS buffer with Tween (PBST) [0.05% Tween 20 in 1× phosphate-buffered saline (PBS)]. The beads were then blocked with 3% BSA in PBST overnight at 4°C. The following day, the beads were suspended in fresh 3% BSA in PBST and blocked for an additional 2 h. The beads were then washed three times with PBST and suspended in 30 µl of 1× PBS to yield a final suspension of blocked magnetic beads.

A dilution series of virus particles ranging from 1:10^3^ to 1:10^7^ was prepared by first taking a 1-µl aliquot of a norovirus GII.4 Sydney positive stool sample and diluting it into 1 ml of PBS. The resulting 1:10^3^ sample was serially diluted by factors of ten into PBS to generate the rest of the dilution series. Biotin-labelled synbody^35^ ASU1052 was then added to a concentration of 1 µM into each diluted sample and incubated with shaking for 1 h at room temperature. The solutions were then added to the blocked streptavidin-coated magnetic beads and shaken for an additional 15 min at room temperature. The beads were washed three times with PBST and one time with PBS and then suspended with 50 µl water. The beads were incubated for 2 min at 95°C to release the viral RNA for analysis. Fifty microliters of each stool dilution was also incubated for 2 min at 95°C and used for comparison.

For cross-reactivity testing and tests of the assay against the GII.6 genotype, 1 µl of GII.4, GII.6 and GI.6 positive stool samples, as well as a norovirus-negative stool sample, were diluted into 1 ml of PBS and followed by the synbody enrichment procedure described above.

## 3. Results and discussion

### 3.1 Design of toehold switches for norovirus GII detection

We first identified conserved sequence regions of the norovirus GII genome suitable for isothermal amplification and toehold switch-based detection. Over 400 norovirus GII complete and partial genome sequences were downloaded from the NCBI database and aligned. A 200-nt target sequence that was highly conserved across the norovirus GII genomes was identified for subsequent amplification and detection experiments. This conserved sequence ran from the C-terminal region of the viral RNA-dependent RNA polymerase through to the N-terminal region of VP1, the major capsid protein.

**Figure 2.**
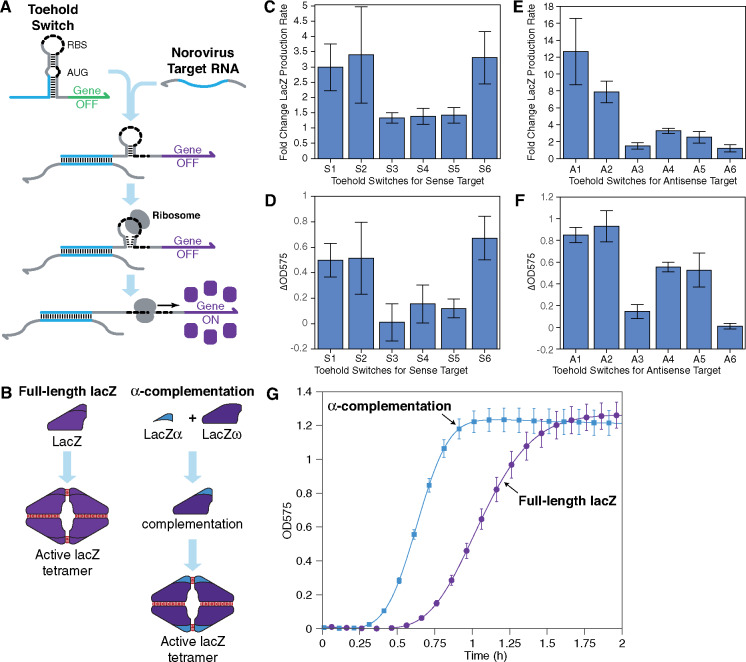
Detection of norovirus target RNA using toehold switches and ɑ-complementation. (**A**) Schematic of toehold switch operation in response to the target RNA. A weak stem containing the ribosomal binding site (RBS) is retained after target binding. This stem unwinds during binding of the ribosome to enable translation of the output gene. (**B**) Enzymatically active lacZ tetramer formation occurs directly for full-length lacZ, while lacZɑ and lacZω must first assemble via ɑ-complementation prior to tetramer formation. (**C**, **D**) Measurements of the fold change in lacZ production rate (C) and ΔOD575 (D) of six toehold switches targeting the sense orientation of the norovirus target RNA. (**E**, **F**) Measurements of the fold change in lacZ production rate (E) and ΔOD575 (F) of six toehold switches targeting the antisense orientation of the norovirus target RNA. Change in lacZ production rate was measured after 1 h of cell-free reaction (C, E) and ΔOD575 was measured after 2 h of cell-free reaction (D, F). (**G**) OD575 for toehold switch A2 as function of cell-free reaction time when outputting full-length lacZ compared to lacZɑ in a reaction supplemented with pre-synthesized lacZω.

Toehold switches for detection of the target sequence were then generated based on an updated design first applied to the detection of the Zika virus. The updated toehold switch design previously provided lower leakage compared to earlier toehold switches^31^ and was originally developed for evaluating AND logic expressions in *E. coli.*^36^ As illustrated in Figure 2a, binding of a cognate target RNA to the updated toehold switch unwinds the lower half of the switch RNA hairpin and leaves the conserved upper stem-loop intact. This upper stem-loop is sufficiently weak to expose the ribosomal binding site (RBS) to enable translation to occur.^36^ Unlike earlier toehold switch mRNA sensors, the updated systems do not employ an RNA refolding domain downstream of the start codon, which could hamper translation of the output gene.

Based on the modified operating mechanism of the toehold switches, we implemented an updated design selection algorithm to identify the toehold switches most likely to be effective at detecting the target RNA. This algorithm modeled the interaction of a series of toehold switches designed to bind along the target RNA in 1-nt increments using the NUPACK software package.^37^^,^^38^ Ensemble defect levels and the affinity of the toehold switch for the target RNA were used to select designs most likely to perform well. Since the target RNA can be transcribed in either the sense or antisense direction following amplification, the top six toehold switches for the sense and antisense target RNAs were selected for experimental testing.

### 3.2 Faster RNA detection with toehold switches using ɑ-complementation of lacZ

In previous work using paper-based cell-free systems, the lacZ enzyme has been used as the output gene for the toehold switch to produce a visible test result through cleavage of a chromogenic substrate.^30^^,^^31^ LacZ, however, at 3.1 kb in length is a relatively long reporter gene compared to alternatives such as green fluorescent protein (0.75 kb) and mCherry (0.72 kb), which leads to several drawbacks. In particular, the longer length of lacZ means that a greater fraction of the cell-free system resources is consumed during transcription and translation, which weakens the output from the assay, and longer times are required for the protein to be synthesized and fold, which increases the time required for the test.

In response to the above limitations, we investigated using ɑ-complementation of lacZ to decrease assay times and strengthen output from the cell-free transcription–translation reactions. Alpha-complementation is a widely applied technique often used for screening cloning vectors. It works by dividing the lacZ enzyme into two peptides termed ɑ and ω (Figure 2B). The lacZ ɑ-peptide (lacZɑ) consists of the first 50 to 59 residues from the N terminus of lacZ and the ω-peptide (lacZω) comprises the remaining ∼970 lacZ residues. The complete lacZ must form a tetramer before it becomes catalytically active; however, lacZω cannot form a tetramer on its own as it lacks residues critical for assembly. As a result, both lacZɑ and lacZω must be expressed before complementation occurs and an active lacZ tetramer can assemble.

We thus implemented toehold switches that used lacZɑ as the output protein and added the much larger lacZω peptide as a pre-synthesized component to the paper-based cell-free reactions. Since lacZɑ is encoded in 180 bp, which is only ∼6% of the length of the full lacZ gene, translation of each lacZɑ molecule should occur faster compared to lacZ and could in principle impose a substantially smaller burden on the cell-free system for each active lacZ tetramer formed. DNA encoding the norovirus-specific toehold switches was cloned into vectors upstream of the lacZɑ open reading frame. Following sequence confirmation, the resulting plasmids were tested in paper-based cell-free reactions supplemented with lacZω, and cleavage of the chromogenic substrate chlorophenol red-b-D-galactopyranoside was monitored using a plate reader. Figure 2C–F shows the results of these experiments with six toehold switches named S1, S2, etc., for the sense orientation of the target RNA and six toehold switches named A1, A2, etc., for the antisense target orientation. All of the toehold switches were tested in parallel with reactions in which no target RNA was present. These experiments were then used to determine the fold change in the lacZ production rate and the ΔOD575 for each sensor. Three of the sense toehold switches provided ON/OFF ratios of approximately three or more (Figure 2C) and displayed a change in absorbance at 575 nm (ΔOD575) of at least 0.4 (Figure 2D), which can be discerned by eye. The toehold switches for the antisense target provided better performance overall with ON/OFF ratios up to 12.6-fold for A1 (Figure 2E) and ΔOD575 up to 0.92 for A2 (Figure 2F). Although the *in silico* selection algorithm successfully generated functional toehold switches for the two norovirus targets, we only detected appreciable correlations between the scoring function and the toehold switches for the antisense target. The sense target devices showed no correlations with the scoring function. Analysis of the experimental data indicate that other combinations of ensemble defect parameters coupled with different weighting factors can provide more accurate predictions of device performance (see Supplementary Information and Supplementary Figure S2).

To determine the effect of ɑ-complementation on detection speed, we took one of the better performing toehold switches, A2, and inserted it into a plasmid upstream of the full lacZ open reading frame. PCR was then used to amplify linear DNA fragments from both lacZɑ and full-length lacZ plasmids and equal concentrations of the two DNA products were tested in paper-based cell-free reactions in the presence of the norovirus target RNA. We observed a substantial increase in the speed of the colorimetric reaction for the lacZɑ systems compared to full-length lacZ (Figure 2G). Applying OD575 = 0.4 as the detection threshold, the lacZɑ reporter reached a positive result in 33 min compared to 56 min for the complete lacZ, which corresponds to a 41% reduction in detection time (see Supplementary Figure S3 for photographs of the paper-based reactions at different OD575 values). Since both reactions reach saturation and completely cleave the substrate within the 2-h measurement shown in Figure 2G, we attribute the increased speed of the reaction in these conditions to the faster folding time of lacZɑ compared to lacZ, rather than to any decrease in the burden on the cell-free reaction caused by the shorter reporter protein.

### 3.3 Isothermal amplification using NASBA and RT-RPA

Since the concentrations of norovirus in stool samples from symptomatic patients range from ∼30 aM to ∼3 pM,^39^ the toehold switches cannot be efficiently activated by viral nucleic acids without an amplification step. We investigated the NASBA and RT-RPA isothermal amplification techniques to determine which provided the lowest limit of detection against the norovirus GII.4 target RNA. The six toehold switches providing the highest ON/OFF ratios were selected for testing with amplified RNA. Since each sensor targeted different regions within the conserved target sequence, we evaluated different amplification primers for each sensor. One primer from each pair contained a 5′ T7 promoter sequence so that the resulting amplicon could be transcribed into RNA for optimal detection using the corresponding toehold switch.

**Figure 3.**
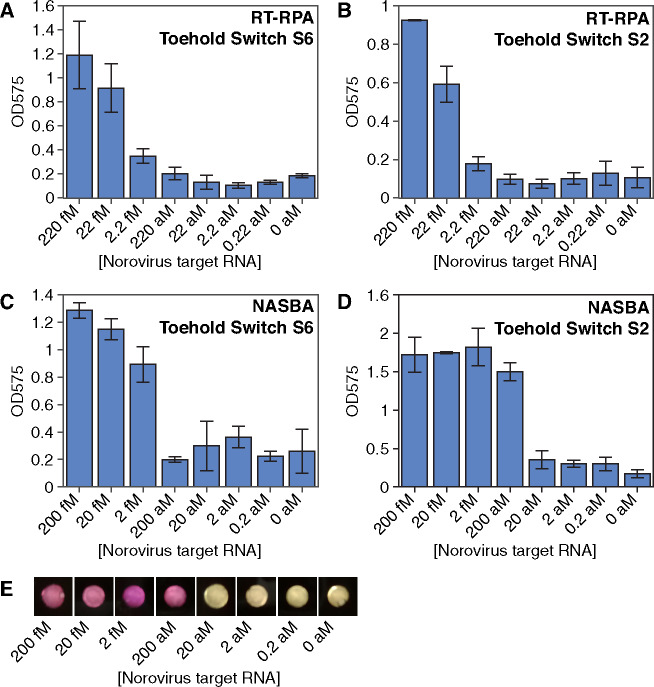
Detection limit measurements for synthetic norovirus GII.4 target RNAs subject to isothermal amplification and detection using toehold switches. (**A, B**) OD575 after amplification using RT-RPA and detection using toehold switches S6 (A) and S2 (B) in 2-h cell-free reactions. (**C, D**) OD575 after amplification using NASBA and detection using toehold switch S6 (C) and S2 (D) in 2-h cell-free reactions. (**E**) Photographs of paper-based reactions using NASBA for amplification and toehold switch S2 for detection. Photographs were taken after 1 h of the cell-free reactions.

Toehold switches S2 and S6 provided the lowest detection limits in the amplification tests. Two-hour amplification reactions were run with synthetic norovirus GII.4 target RNAs ranging in concentration from 220 fM to 0.2 aM. The amplified products were then diluted 7-fold and applied to the toehold switch reactions. For the RT-RPA reactions, both S2 and S6 toehold switches could detect down to 22 fM of the norovirus RNA with colorimetric outputs that could be readily discerned by eye (Figure 3A, B). Statistically significant concentrations as low as 2.2 fM could be detected from quantitative plate reader absorbance measurements for toehold switch S2 after 3 h and toehold switch S6 after 1 h.

NASBA tests provided improved detection limits compared to RPA. For toehold switch S6, we could discern concentrations down to 2 fM by eye within 2 h and by plate reader within 1 h (Figure 3C). Although toehold switch S2 was not one of the very top performers in the initial screen (Figure 2), it provided the lowest detection limit when coupled with NASBA. Experiments showed this sensor could detect down to 200 aM concentrations of the synthetic norovirus transcript (Figure 3D). In addition, the sensor enabled detection by eye in 60 min at the 200 aM detection limit as shown in Figure 3E and by plate reader in 28 min. A concentration of 200 aM corresponds to 600 copies of the RNA template in the 5 µl NASBA reaction.

### 3.4 Diagnostic validation with active norovirus

**Figure 4.**
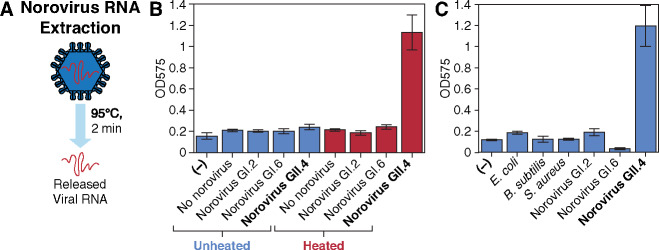
Detection of live norovirus GII.4 Sydney and cross-reactivity testing. (**A**) Norovirus RNA was extracted by diluting a stool sample 1:50 into PBS and briefly heating to 95°C for 2 min. (**B**) Measurement of OD575 after a 2-h paper-based reaction for a water-only negative control (–) and stool samples with and without norovirus particles before and after the brief heating treatment. All samples were subject to amplification via NASBA and detection with toehold switch S2. Only the heated norovirus GII.4 Sydney sample activates the toehold switch. (**C**) Cross-reactivity testing of the assay against RNA from multiple bacteria, norovirus genotypes and a water-only negative control. All samples were subject to NASBA and toehold switch S2 detection. OD575 was measured after 2 h of the cell-free reaction.

To validate the detection platform, we performed experiments with active norovirus samples and tested the assay for cross-reactivity against other potential pathogens. Following previous reports on norovirus^26^^,^^40^ and our earlier work on the Zika virus,^31^ we first evaluated a simple method for extracting viral RNA from infected stool samples using a brief heating step. A norovirus GII.4 Sydney positive stool sample was diluted 1:50 in PBS and heated for 2 min at 95°C (Figure 4A). The same procedure was applied to a stool sample not infected with the virus and two additional stool samples containing norovirus GI.2 and GI.6. These heated samples, along with comparison unheated samples and a water-only negative control, were both amplified by NASBA over 2 h and applied to a paper-based reaction with toehold switch S2. The unheated samples all yielded minimal changes in toehold switch output compared to the negative control. The OD575 of the heated sample with norovirus GII.4 Sydney increased to 1.13, while the OD575 of the other heated samples remained below 0.25 (Figure 4B). Thus, the simple heating method was effective at releasing RNA from norovirus particles and the assay was specific for norovirus GII.4 Sydney.

To further evaluate cross-reactivity, we extracted RNA from *E. coli*, *B. subtilis* and a MRSA strain and added the RNA at masses of 80.6 ng, 123.5 ng and 100.8 ng, respectively, to the NASBA reaction. RNA was also extracted from stool samples containing norovirus GII.4 Sydney, GI.2 and GI.6 and added to the NASBA reaction at a concentration of ∼20 fM. None of these samples of bacterial RNA nor the GI.2 and GI.6 norovirus genotypes were able to activate toehold switch S2 for visual detection. The system was strongly activated by norovirus GII.4 Sydney RNA (Figure 4C).

### 3.5 Norovirus enrichment using a synbody-based magnetic bead technique

**Figure 5.**
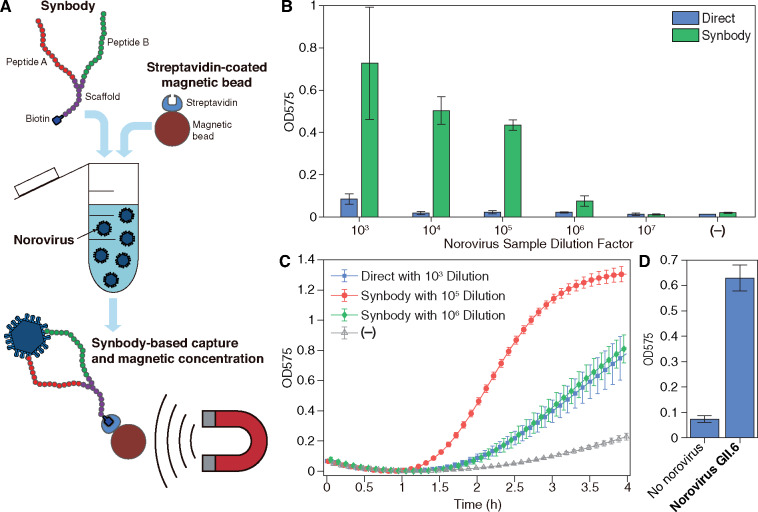
Implementation of a synbody-based capture and concentration method for norovirus detection. (**A**) Illustration of the synbody enrichment technique. Biotin-labelled synbodies engineered to recognize diverse norovirus genotypes are used to bind to virus particles in a dilute solution and are in turn captured by streptavidin-coated magnetic beads. Magnetic capture enables concentration of the captured norovirus particles. (**B**) Measurement of OD575 after 2-h cell-free reactions with toehold switch S2. Samples subject to synbody-based concentration and samples used directly without concentration were amplified by NASBA. The negative control (−) is a water-only sample. (**C**) Time-course measurements of OD575 for synbody-concentrated samples compared to samples used directly. OD575 for a sample used directly after 1000-fold dilution is comparable to a concentrated sample initially diluted by 10^6^-fold. (**D**) Detection of norovirus GII.6 from a stool sample using toehold switch S2 and updated NASBA primers for the GII.6 genome. OD575 measurements were taken after 2 h of the paper-based cell-free reaction and using a norovirus-negative stool sample as comparison.

The ability to identify norovirus in dilute solutions or from large solution volumes is valuable for improving diagnostic sensitivity and for confirming complete decontamination of an area following an outbreak. For instance, dilute liquids, such as cleaning solutions from kitchen and bathroom surfaces, can be tested for residual virus following cleanup. To this end, we employed a synbody-based magnetic bead capture assay to concentrate norovirus from dilute solutions (Figure 5A). Synbodies are synthetic bivalent affinity ligands composed of two 15- to 20-mer peptides screened to bind to the surface of a protein of interest. Synbodies have affinities and specificities similar to antibodies.^41^^,^^42^ Unlike antibodies, however, which often lose their affinity as norovirus strains evolve,^1^ synbodies have broad cross-affinity for multiple norovirus genotypes, which enables them to recognize a range of norovirus genotypes within both the GI and GII genogroups.^35^

To capture and concentrate the virus, we took a stool sample positive for norovirus GII.4 Sydney at a concentration of 270 fM as determined by qRT-PCR and prepared a series of higher dilutions ranging from 1:10^3^ to 1:10^7^ in PBS. Biotin-labelled synbody ASU1052, which was previously validated against multiple norovirus strains,^35^ and streptavidin-coated magnetic beads were added sequentially to the diluted samples with shaking at room temperature for 75 min total. After magnetic capture and washing, the beads were suspended with 50 µl of water and heated to 95°C for 2 min to release the virus RNA. These virus samples, along with comparison ones heated but not subjected to synbody capture, were then amplified using NASBA and applied to paper-based cell-free systems containing toehold switch S2.

Figure 5B displays the absorbance change produced from the reactions after 2 h with the two different sets of samples. For synbody-concentrated samples, norovirus could be detected by eye with dilution factors up to 10^5^, which corresponds to a concentration of 2.7 aM. In contrast, none of the samples used directly and not subjected to concentration could be detected within 2 h by eye. To further compare the two preparation methods, Figure 5C shows the absorbance change over time for several virus samples. The synbody-concentrated sample prepared from a 10^5^ dilution crosses the eye-based detection threshold of OD575 = 0.4 in under 2 h and provides a statistically significant positive signal in the plate reader after 66 min. The profile of the non-concentrated sample diluted 1000-fold nearly matches that of the synbody-concentrated sample diluted 10^6^-fold over the full 4-h measurement. Both samples cross the visual detection threshold after 3 h and provide positive results from quantitative plate reader measurements in approximately 2 h, which correspond to norovirus GII.4 Sydney detection limits of 270 aM and 270 zM for the non-concentrated and synbody-concentrated samples, respectively. The synbody-based concentration technique thus enables a 1000-fold improvement in the detection limit of the norovirus assay.

To determine if the assay could also be applied to closely related norovirus genotypes, we also tested the systems against a stool sample with the norovirus GII.6 genotype. Virus particles were enriched using the ASU1052 synbodies and subject to NASBA using the primers optimized for GII.4 Sydney amplification. Unfortunately, these primers were not effective for this genotype. Primers modified to match the GII.6 genome, however, enabled successful amplification. Despite the presence of some mismatches between toehold switch S2 and its binding site on the GII.6 amplicon (see Supplementary Figure S4), a visible OD575 signal was observed from paper-based reactions within 2 h (Figure 4D). Thus, toehold switch S2 is capable of detecting amplicons from both the norovirus GII.4 Sydney and GII.6 genotypes.

## 4. Conclusions

We have demonstrated a paper-based assay for detection of norovirus that does not require expensive thermal cycling equipment, provides test results that can be read directly by eye, and employs toehold switch riboregulators to eliminate false positives caused by non-specific amplification. The assay enables visual detection of norovirus down to a concentration of 270 aM from clinical stool samples containing live norovirus particles from the GII.4 Sydney genotype. The addition of a virus capture and concentration step using synbodies enables a further 1000-fold improvement in the sensitivity of the assay, allowing concentrations as low as 270 zM to be detected by eye after a 3-h paper-based reaction. This work also demonstrates that paper-based transcription–translation systems can remain active upon exposure to samples diluted from stool and confirms that RPA products can be successfully detected in the cell-free reactions, albeit with a higher detection limit than comparison NASBA products.

The norovirus assay provides significant improvements in sensitivity compared to our previously reported diagnostic assay for the Zika virus.^31^ The Zika virus test provided a 1 fM detection limit against synthetic target RNAs and detected the virus from plasma at a concentration of 2.8 fM. In contrast, the norovirus assay demonstrated a 5-fold lower detection limit of 200 aM against a synthetic target and was successfully applied to a stool sample with a 270 aM concentration of norovirus. Addition of the synbody concentration step thus yielded an overall 5000-fold improvement in the detection limit. The Zika virus is known to be present at very low levels in symptomatic patients, with serum concentrations ranging from 8 zM to 6.1 fM with an average of 160 aM.^43^ These concentrations are 10- to 100-fold lower than those observed for patients with the related dengue and chikungunya viruses.^44^ Accordingly, our synbody-based concentration methods could prove valuable for extending the existing Zika test to more carriers of the virus. While the Zika diagnostic was only applied to a plasma sample from a viremic rhesus macaque, we have also demonstrated in this work that the diagnostic platform can be used on human stool samples, which can be used to identify many other causes of acute gastrointestinal illness beyond norovirus.

Although our norovirus assay provides sufficient sensitivity for detection from clinical samples, at present it requires 3–6 h of processing time to reach a test result, which is substantially longer than many other diagnostics that employ isothermal amplification. We expect that large reductions in assay time can be obtained by further optimization of the synbody-based enrichment technique, by designing toehold switches optimized for quicker and stronger output, and by implementing new reporter proteins with faster activation. Indeed, the substantial decrease in reaction time that we observed using ɑ-complementation of lacZ suggests that there is ample room for improvement using alternative reporters. Moreover, use of faster amplification techniques such as RT-RPA with improved primers or strand-displacement amplification could further decrease the time to detection for the technique. We also expect that toehold switch dynamic range against pathogen RNAs can be improved with continued refinement of *in silico* selection algorithms. In particular, screening experiments examining larger numbers of toehold switches against diverse target RNAs will be essential for generating *in silico* design scoring functions that are able to accurately predict their performance when deployed in cell-free transcription–translation systems.

The assay can also be improved by reducing its cost. In addition to the ∼$1/test price of the paper-based component of the assay,^30^ the per test costs of NASBA, streptavidin-coated magnetic beads, and biotinylated synbodies are $2.25, $5.38 and $0.10, respectively. The total cost in materials for the assay is thus $8.73 and the overall assay requires approximately 35 min of hands on time. A previous study in South Africa to assess GeneXpert cartridge costs has reported an average lab technician salary of $9.07/h,^14^ which brings the total assay cost to $14.02 with labor included. Materials costs for this estimate are based on retail prices for the components. It is likely that the quantities of magnetic beads used in the assay can be reduced substantially with further refinement of the experimental procedures, and materials costs can decrease with purchases at larger scales. Even without optimization of the assay toward reduced price, the total cost per assay remains lower than the $14.93 calculated for GeneXpert cartridges in South Africa where concessional pricing is in effect.^14^ Furthermore, our assay does not require large initial expenditures for purchasing expensive equipment.

The continual emergence of new variants of norovirus means that our paper-based assay will need to be updated as other strains replace GII.4 Sydney to ensure that false negatives do not occur. For instance, the GII.P17-GII.17 norovirus strain has recently become predominant in Asia^10^ and immunochromatographic tests, which were developed for the GII.4 strain, have demonstrated 1000-fold poorer detection limits against the emergent strain.^45^ To reduce the probability of false negatives, our assay employs a target sequence that is well conserved across different GII strains, including GII.P17 and GII.17. The toehold switch S2 sensor is predicted by NUPACK simulations to tolerate several mismatches in the target RNA, particularly within the toehold region, and still expose the RBS and start codon to enable translation of the reporter gene (see Supplementary Figure S4). This resiliency against sequence variations is evidenced by the ability of device S2 to activate against the GII.6 strain (Figure 5D). In cases where there is larger sequence divergence, sensor mRNAs that employ multiple toehold switch hairpins upstream of a single output gene can be used to detect different norovirus strains or to compensate for locations with higher sequence variability to avoid false negatives. We have demonstrated that such OR logic systems can be used to detect six completely sequence-independent target RNAs using a single sensor mRNA in *E. coli.*^36^ We expect that similar approaches can be used in the paper-based reactions and prove more parsimonious with cell-free systems resources than other implementations employing multiple independent mRNAs. Like other nucleic acid tests that employ amplification, false negatives can also occur when the amplification primers do not have sufficient homology with the target amplicon. Such sequence variability can be addressed using primers with degenerate bases at positions known to have high probability of sequence divergence.

Despite these areas for improvement, the reasonably low cost of the assay and its reliance on only inexpensive equipment enables it to be implemented in decentralized contexts such as remote clinics or cruise ships with trained operators. Furthermore, coupling the validated molecular components of the assay with companion hardware for incubation and readout^31^ or liquid handling^46^ has the potential to substantially reduce operator training requirements and lead to more widespread deployment in the future. Lastly, the demonstrated ability of synbodies and toehold switches to bind to proteins and nucleic acids, respectively, from a variety of different pathogens^30–32^^,^^41^^,^^42^ indicates that our combined concentration and detection approach can be successfully applied to a diverse range of infectious agents.

## Supplementary Material

Supplementary Data
