# Supplementary material for: Low-cost detection of norovirus using paper-based cell-free systems and synbody-based viral enrichment
Source: Synth Biol (Oxf). 2018 Sep 19;3(1):ysy018. doi: 10.1093/synbio/ysy018 (PMC6195790; doi:10.1093/synbio/ysy018)
Supplement: Supplementary Data [file ysy018_supp.zip › GREEN_norovirus_SI.docx]

**Supplementary data for:**

**Low-Cost Detection of Norovirus Using Paper-Based Cell-Free Systems and Synbody-Based Viral Enrichment**

Duo Ma^1^, Luhui Shen^2^, Kaiyue Wu^1^, Chris W. Diehnelt^2^, Alexander A. Green*^1^

^1^Biodesign Center for Molecular Design and Biomimetics and ^2^Biodesign Center for Innovations in Medicine, The Biodesign Institute and School of Molecular Sciences, Arizona State University, AZ 85287, USA

**Supplemental Data Methods**

**Calculation of Ensemble Defects for Toehold Switch Designs**

Ensemble defect levels were calculated using NUPACK for toehold switch designs over the regions specified in the main text and indicated in Supplementary Figure S1. The toehold switch designs for norovirus contained a conserved upper hairpin domain with the sequence GGACUUUAGAAC**AGAGGAGA**UAAAG**AUG**, with the RBS and start codon shown in bold, and a 31-nt linker between the sensor and the output gene with the sequence AACCUGGCGGCAGCGCAAGAAGAUGCGUAAA. The parameters *d*_min_sensor_ and *d*_toehold_ were calculated by first computing the pairwise binding probabilities for the toehold switch sequence from the 5’ end through to the 31st base beyond the 3’ end of the hairpin (i.e. the full sequence shown in Supplementary Figure S1). These binding probabilities were then used to compute the ensemble defect from the specified sequence regions of *d*_min_sensor_ and *d*_toehold_ using the target secondary structures shown in Supplementary Figure S1. Calculating the ensemble defect in this way enabled the effect of sequences outside the main region of interest to be considered for design purposes. For *d*_binding_site_, the ensemble defect was calculated in an analogous manner using the pairwise binding probabilities of the complete target RNA sequence and specifying a completely single-stranded ideal secondary structure in the binding site region. For *d*_active_sensor_, the ensemble defect was calculated directly from the sequence region starting from the base indicated in Supplementary Figure S1. A completely single-stranded secondary structure was used for assessing design quality for *d*_active_sensor_.

In addition to the four terms above, we calculated two additional ensemble defect parameters during the design process. The term *d*_full_sensor_ was generated by computing the ensemble defect for the full toehold switch sequence and structure shown in Supplementary Figure S1. The term *d*_min_target_ was generated by taking the 36-nt sequence targeted by the toehold switch and computing its ensemble defect with a completely single-stranded ideal structure.

**Assessment and Further Optimization of Toehold Switch Selection Algorithm**

To determine the effectiveness of the described toehold switch selection method, we have taken experimental fold change in lacZ production data (Figure 2C,E) and investigated if these data display significant correlations with the selection scoring function and the six computed ensemble defect parameters. Correlations between these parameters and the experimental results were assessed using R^2^ values and regression coefficients generated from the Matlab multiple linear regression using least squares function regress. For these regressions, a column of ones was appended to the matrix of predictor variables to allow the model to include a constant term or offset.

Supplementary Figure S2A shows the linear regression obtained using experimental fold change from the toehold switches for the antisense target and the design scoring function *s*. We found that the scoring function, which made use of four ensemble defect parameters, provided a fairly strong correlation with the experimental data with R^2^ = 0.62. We then examined the correlations between the antisense experimental data and the set of six ensemble defect parameters. The two parameters that showed the strongest correlations were *d*_active_sensor_ and *d*_min_target_, which yielded R^2^ of 0.52 and 0.31, respectively (see Supplementary Figure S2B-C). A two-parameter linear regression combining both these terms provided R^2^ = 0.62, which matched the correlation observed for the four-parameter scoring function.

We applied the same series of analyses to the set of toehold switches for the sense norovirus target. However, these devices showed much weaker correlations between design parameters and experimental results (see Supplementary Figure S2E-H). The scoring function did not display any correlation with the fold change in lacZ (R^2^ = 0.07) and the top two single-parameter fits did not display strong correlations, R^2^ = 0.26 and R^2^ = 0.20 for *d*_binding_site_ and *d*_active_sensor_, respectively. Furthermore, no improvements were observed by combining the two parameters into the same regression.

Since the terms used in the scoring function were originally normalized for each target RNA, we could not use the scoring function directly to determine if it was highly correlated with the experimental results from all 12 devices since they bound to different target RNAs. Instead, we took the fold change experimental results and supplied the regression with the set of the four predictor variables used by the scoring function but in non-normalized form: *d*_min_sensor_, *d*_toehold_, *d*_binding_site_, and *d*_active_sensor_. (Note: The fifth predictor variable *f*, the equilibrium fraction, used in the scoring function was equal to one for all devices tested.). In this case, the linear regression provided limited correlation with R^2^ = 0.29 (see Supplementary Figure S2I). To determine if other combinations of ensemble defect parameters could provide a stronger correlation with the experimental data, we computed linear regressions for all two-, three-, and four-parameter combinations as shown in Supplementary Figure S2J-L. We found that the combination of *d*_binding_site_ and *d*_active_sensor_ were most effective for the two-parameter fits, yielding R^2^ = 0.42_,_ a substantial increase over the scoring function combination. Addition of *d*_full_sensor_ to the pair provided the best three-parameter fit with another sizeable increase in fit quality to R^2^ = 0.57. Finally, the optimal four-parameter fit, which added *d*_min_target_ to the trio of predictor variables, provided a small increase in R^2^ to 0.60.

The three- and four-parameter linear regressions generated the following equations for predicting the fold change for the toehold switch sensors:

*Three-parameter fit (R^2^ = 0.57):*

Fold change = –71.7 *d*_full_sensor_  – 49.1 *d*_active_sensor_ – 22.6 *d*_binding_site_ + 54.3

*Four-parameter fit (R^2^ = 0.60):*

Fold change = –93.2 *d*_full_sensor_ – 43.3 *d*_active_sensor_ – 22.1 *d*_binding_site_ – 9.4 *d*_min_target_ + 61.3

For both these linear fitting functions, negative coefficients are used in front of all of the ensemble defect parameters as expected, since lower defect levels should lead to higher toehold switch performance (i.e. fold change). In addition, the parameters *d*_full_sensor_, *d*_active_sensor_, and *d*_binding_site_ are listed from highest to lowest fitting function weighting factor. These three parameters define the most critical functional elements of the toehold switch devices. A properly folded secondary structure of the full sensor is required to provide a toehold region for target binding and a strong hairpin structure to repress translational leakage. The active sensor requires a translation start site with low secondary structure to promote rapid production of the output gene. Lastly, a binding site with low secondary structure helps ensure that the site is accessible for sensor binding. We expect that design selection algorithms can be further improved using strategies similar to the one described here and using much larger libraries of toehold switches to probe a wider range of sensor and target sequences experimentally.

**Supplementary Table 1. Toehold switch and norovirus target RNA sequences**

| **Name** | **RNA Sequence** |
| --- | --- |
| Toehold switch S1 RNA | GGGCCAUCUUCAUUCACAAAACUGGGAGCCAGAUUGCGAGGACUUUAGAACAGAGGAGAUAAAGAUGUCGCAAUCUGGAACCUGGCGGCAGCGCAAGAAGAUG |
| Toehold switch S2 RNA | GGGAUCGCCCUCCCACGUGCUCAGAUCUGAGAAUCUCAUGGACUUUAGAACAGAGGAGAUAAAGAUGAUGAGAUUCUCAACCUGGCGGCAGCGCAAGAAGAUG |
| Toehold switch S3 RNA | GGGACAAAACUGGGAGCCAGAUUGCGAUCGCCCUCCCACGGACUUUAGAACAGAGGAGAUAAAGAUGGUGGGAGGGCGAACCUGGCGGCAGCGCAAGAAGAUG |
| Toehold switch S4 RNA | GGGCUGGGACGAGGUUGGCUGCGGACCCAUCAGAUGGGUGGACUUUAGAACAGAGGAGAUAAAGAUGACCCAUCUGAUAACCUGGCGGCAGCGCAAGAAGAUG |
| Toehold switch S5 RNA | GGGUCAUUCGACGCCAUCUUCAUUCACAAAACUGGGAGGGACUUUAGAACAGAGGAGAUAAAGAUGCUCCCAGUUUUAACCUGGCGGCAGCGCAAGAAGAUG |
| Toehold switch S6 RNA | GGGAGCCAGAUUGCGAUCGCCCUCCCACGUGCUCAGAUCGGACUUUAGAACAGAGGAGAUAAAGAUGGAUCUGAGCACAACCUGGCGGCAGCGCAAGAAGAUG |
| Toehold switch A1 RNA | GGGUCUGAUGGGUCCGCAGCCAACCUCGUCCCAGAGGUCGGACUUUAGAACAGAGGAGAUAAAGAUGGACCUCUGGGAAACCUGGCGGCAGCGCAAGAAGAUG |
| Toehold switch A2 RNA | GGGUGGGAGGGCGAUCGCAAUCUGGCUCCCAGUUUUGUGGACUUUAGAACAGAGGAGAUAAAGAUGACAAAACUGGGAACCUGGCGGCAGCGCAAGAAGAUG |
| Toehold switch A3 RNA | GGGUGUGAAUGAAGAUGGCGUCGAAUGACGCCAACCCAUGGACUUUAGAACAGAGGAGAUAAAGAUGAUGGGUUGGCGAACCUGGCGGCAGCGCAAGAAGAUG |
| Toehold switch A4 RNA | GGGAGAUCUGAGCACGUGGGAGGGCGAUCGCAAUCUGGCGGACUUUAGAACAGAGGAGAUAAAGAUGGCCAGAUUGCGAACCUGGCGGCAGCGCAAGAAGAUG |
| Toehold switch A5 RNA | GGGAUCGCAAUCUGGCUCCCAGUUUUGUGAAUGAAGAUGGGACUUUAGAACAGAGGAGAUAAAGAUGCAUCUUCAUUCAACCUGGCGGCAGCGCAAGAAGAUG |
| Toehold switch A6 RNA | GGGUCGAAUGACGCCAACCCAUCUGAUGGGUCCGCAGCCGGACUUUAGAACAGAGGAGAUAAAGAUGGGCUGCGGACCAACCUGGCGGCAGCGCAAGAAGAUG |
| Norovirus GII.4 sense target | AUGGAUUUUUACGUGCCCAGGCAAGAGCCAAUGUUCAGAUGGAUGAGAUUCUCAGAUCUGAGCACGUGGGAGGGCGAUCGCAAUCUGGCUCCCAGUUUUGUGAAUGAAGAUGGCGUCGAAUGACGCCAACCCAUCUGAUGGGUCCGCAGCCAACCUCGUCCCAGAGGUCAACAAUGAGGUUAUGGCUUUGGAGCCCGU |
| Norovirus GII.4 antisense target | ACGGGCUCCAAAGCCAUAACCUCAUUGUUGACCUCUGGGACGAGGUUGGCUGCGGACCCAUCAGAUGGGUUGGCGUCAUUCGACGCCAUCUUCAUUCACAAAACUGGGAGCCAGAUUGCGAUCGCCCUCCCACGUGCUCAGAUCUGAGAAUCUCAUCCAUCUGAACAUUGGCUCUUGCCUGGGCACGUAAAAAUCCAU |
| Norovirus GII.P17 sense target | AUGGAUUUUUAUGUGCCCAGACAAGAGUCAAUGUUCAGAUGGAUGAGGUUCUCAGAUCUAAGCACAUGGGAGGGCGAUCGCAAUCUGGCUCCCAGUUUUGUGAAUGAAGAUGGCGUCGAAUGACGCCGCUCCAUCUAAUGAUGGUGCUGCUGGUCUCGUACCAGAGGGCAACAACGAG |
| Norovirus GII.17 sense target | AUGGAUUUUUAUGUGCCCAGACAAGAGUCAAUGUUCAGAUGGAUGAGGUUCUCAGAUCUAAGCACAUGGGAGGGCGAUCGCAAUCUGGCUCCCAGUUUUGUGAAUGAAGAUGGCGUCGAAUGACGCCGCUCCAUCUAAUGAUGGUGCUGCUGGUCUCGUACCAGAGGGCAACAACGAG |
| Norovirus GII.6 antisense target | UGGAGUUUUAUGUGCCCAGACAAGAGGCCAUGUUCAGGUGGAUGAGAUUCUCUGACCUCAGCACAUGGGAGGGCGAUCGCAAUCUUGCUCCCGAGGGUGUGAAUGAAGAUGGCGUCGAAUGACGCUGCUCCAUCGAAUGAUGGUGCUGCCAACCUCGUACCAGAGGCCAACAAUGAGGUUAUGGC |

**Supplementary Table 2. List of Plasmids**

| **Name** | **Marker** | **Description** |
| --- | --- | --- |
| pAT_T7_HisLacZ | Amp | T7 RNAP-driven expression of N-terminal His-tagged lacZ. pET15b backbone. |
| ZIKV_Sensor_27B_LacZ (Addgene #: 75006) | Kan | T7 RNAP-driven expression of Zika virus sensing toehold switch with lacZ reporter. pCOLAduet backbone. |
| pDM_T7_HisLacZomega | Amp | T7 RNAP-drive expression of N-terminal His-tagged lacZω. pET15b backbone. |
| pDM_noro_S1_lacZA | Kan | T7 RNAP-driven expression of norovirus sense orientation toehold switches (S1 to S6) with a lacZɑ reporter. pCOLAduet backbone. |
| pDM_noro_S2_lacZA |  |  |
| pDM_noro_S3_lacZA |  |  |
| pDM_noro_S4_lacZA |  |  |
| pDM_noro_S5_lacZA |  |  |
| pDM_noro_S6_lacZA |  |  |
| pDM_noro_A1_lacZA | Kan | T7 RNAP-driven expression of norovirus antisense orientation toehold switches (A1 to A6) with a lacZɑ reporter. pCOLAduet backbone. |
| pDM_noro_A2_lacZA |  |  |
| pDM_noro_A3_lacZA |  |  |
| pDM_noro_A4_lacZA |  |  |
| pDM_noro_A5_lacZA |  |  |
| pDM_noro_A6_lacZA |  |  |
| pDM_noro_A2_lacZ | Kan | T7 RNAP-driven expression of norovirus antisense orientation toehold switch A2 with full-length lacZ reporter. pCOLAduet backbone. |

**Supplementary Table 3. List of PCR Primers Used for Plasmid Construction**

| **Primer Name** | **Sequence** | **DNA template** | **Destination plasmid(s)** |
| --- | --- | --- | --- |
| lacZ_pET15b_fwd | TAACTAGCATAACCCCTTGGGG | pET15b | pAT_T7_HisLacZ |
| lacZ_pET15b_rev | CATATGGCTGCCGCGCGG |  |  |
| lacZ_insert_fwd | AGCGGCCTGGTGCCGCGCGGCAGCCATATGCGTAAAATGACCATGATTACGGATTCACT | *E. coli* MG1655 genome |  |
| lacZ_insert_rev | TTTAGAGGCCCCAAGGGGTTATGCTAGTTATTTTTGACACCAGACCAACTGGT |  |  |
| lacZomega_BB_fwd | AACAGTTGCGCAGCCTGA | pAT_T7_HisLacZ | pDM_T7_HisLacZomega |
| lacZomega_BB_rev | CCAGTGAATCCGTAATCATGGTCAT |  |  |
| lacZomega_insert_L | ATGACCATGATTACGGATTCACTGGCCGTCGCCCGCACCGA | lacZomega_insert_R |  |
| lacZomega_insert_R | TCAGGCTGCGCAACTGTTGGGAAGGGCGATCGGTGCGGGC | lacZomega_insert_L |  |
| lacZalpha_BB_fwd | TAGCATAACCCCTTGGGGC | pDM_noro_A2_lacZA | pDM_noro_A2_lacZA |
| lacZalpha_BB_rev | GCGCAACTGTTGGGAAGG |  |  |
| lacZalpha_insert_L | CGCACCGATCGCCCTTCCCAACAGTTGCGCAGCCTGAATGGCGAATGGTAAT | lacZalpha_insert_R |  |
| lacZalpha_insert_R | CCCGTTTAGAGGCCCCAAGGGGTTATGCTATTATTACCATTCGCCATTCAGG | lacZalpha_insert_L |  |
| lacZ_BB_fwd | ATGACCATGATTACGGATTCACTGGCCGTC | ZIKV_Sensor_27B_LacZ, pDM_noro_A2_lacZA | pDM_noro_A#_lacZ, pDM_noro_A#_lacZA, pDM_noro_S#_lacZ, pDM_noro_S#_lacZA, where # = {1,2,3,4,5,6} |
| Dstar_lacZ_BB_rev | CCGGCTACCGTAGAAACGCGAATTTACTAGCATAAGGGAGAGCGTCGAGATC |  |  |
| Dnorm_TS_insert_fwd | CTAGTAAATTCGCGTTTCTACGGTAGCCGGGCGCTAATACGACTCACTATAGGG | Toehold switch DNA strands |  |
| TS_insert_linker_rev | GACGGCCAGTGAATCCGTAATCATGGTCATCTTCTTGCGCTGCCGCCAGGTT |  |  |

**Supplementary Table 4. List of DNA Strands Used for Toehold Switches and Sequencing**

| **Primer Name** | **Sequence** | **Description** |
| --- | --- | --- |
| Toehold switch S1 DNA | GCGCTAATACGACTCACTATAGGGCCATCTTCATTCACAAAACTGGGAGCCAGATTGCGAGGACTTTAGAACAGAGGAGATAAAGATGTCGCAATCTGGAACCTGGCGGCAGCGCAAGAAGATG | Toehold switch DNA templates |
| Toehold switch S2 DNA | GCGCTAATACGACTCACTATAGGGATCGCCCTCCCACGTGCTCAGATCTGAGAATCTCATGGACTTTAGAACAGAGGAGATAAAGATGATGAGATTCTCAACCTGGCGGCAGCGCAAGAAGATG |  |
| Toehold switch S3 DNA | GCGCTAATACGACTCACTATAGGGACAAAACTGGGAGCCAGATTGCGATCGCCCTCCCACGGACTTTAGAACAGAGGAGATAAAGATGGTGGGAGGGCGAACCTGGCGGCAGCGCAAGAAGATG |  |
| Toehold switch S4 DNA | GCGCTAATACGACTCACTATAGGGCTGGGACGAGGTTGGCTGCGGACCCATCAGATGGGTGGACTTTAGAACAGAGGAGATAAAGATGACCCATCTGATAACCTGGCGGCAGCGCAAGAAGATG |  |
| Toehold switch S5 DNA | GCGCTAATACGACTCACTATAGGGTCATTCGACGCCATCTTCATTCACAAAACTGGGAGGGACTTTAGAACAGAGGAGATAAAGATGCTCCCAGTTTTAACCTGGCGGCAGCGCAAGAAGATG |  |
| Toehold switch S6 DNA | GCGCTAATACGACTCACTATAGGGAGCCAGATTGCGATCGCCCTCCCACGTGCTCAGATCGGACTTTAGAACAGAGGAGATAAAGATGGATCTGAGCACAACCTGGCGGCAGCGCAAGAAGATG |  |
| Toehold switch A1 DNA | GCGCTAATACGACTCACTATAGGGTCTGATGGGTCCGCAGCCAACCTCGTCCCAGAGGTCGGACTTTAGAACAGAGGAGATAAAGATGGACCTCTGGGAAACCTGGCGGCAGCGCAAGAAGATG |  |
| Toehold switch A2 DNA | GCGCTAATACGACTCACTATAGGGTGGGAGGGCGATCGCAATCTGGCTCCCAGTTTTGTGGACTTTAGAACAGAGGAGATAAAGATGACAAAACTGGGAACCTGGCGGCAGCGCAAGAAGATG |  |
| Toehold switch A3 DNA | GCGCTAATACGACTCACTATAGGGTGTGAATGAAGATGGCGTCGAATGACGCCAACCCATGGACTTTAGAACAGAGGAGATAAAGATGATGGGTTGGCGAACCTGGCGGCAGCGCAAGAAGATG |  |
| Toehold switch A4 DNA | GCGCTAATACGACTCACTATAGGGAGATCTGAGCACGTGGGAGGGCGATCGCAATCTGGCGGACTTTAGAACAGAGGAGATAAAGATGGCCAGATTGCGAACCTGGCGGCAGCGCAAGAAGATG |  |
| Toehold switch A5 DNA | GCGCTAATACGACTCACTATAGGGATCGCAATCTGGCTCCCAGTTTTGTGAATGAAGATGGGACTTTAGAACAGAGGAGATAAAGATGCATCTTCATTCAACCTGGCGGCAGCGCAAGAAGATG |  |
| Toehold switch A6 DNA | GCGCTAATACGACTCACTATAGGGTCGAATGACGCCAACCCATCTGATGGGTCCGCAGCCGGACTTTAGAACAGAGGAGATAAAGATGGGCTGCGGACCAACCTGGCGGCAGCGCAAGAAGATG |  |
| pCOLA_seq_fwd | CGTTACTGGTTTCACATTCACCACCC | Sequencing primer used for confirming sequence of toehold switch sensors inserted in pCOLA, pCDF, pACYC expression vectors. |
| pET15b_seq_fwd1 | CCTGCCACCATACCCACGC | Sequencing primer used for confirming sequence of genes inserted into pET15b vectors within the multiple cloning site region. |

**Supplementary Table 5. Norovirus isothermal amplification primers**

| **Norovirus Genotype** | **Toehold Switch** | **Forward Primer** | **Reverse Primer** |
| --- | --- | --- | --- |
| GII.4 | S1 | AATTCTAATACGACTCACTATAGGGAGAAGGATTCTCAGATCTGAGCACGTGGGA | ATTGTTGACCTCTGGGACGA |
|  | S2 | AATTCTAATACGACTCACTATAGGGAGAAGGCAGGCAAGAGCCAATGTTCAGA | CTCATTGTTGACCTCTGGGA |
|  | S6 | AATTCTAATACGACTCACTATAGGGAGAAGGGCAAGAGCCAATGTTCAGATGGA | CTCATTGTTGACCTCTGGGA |
|  | A1 | AATTCTAATACGACTCACTATAGGGAGAAGGGCTCCAAAGCCATAACCTCA | GCAAGAGCCAATGTTCAGATGGA |
|  | A2 | AATTCTAATACGACTCACTATAGGGAGAAGGCTCATTGTTGACCTCTGGGA | GATGGATGAGATTCTCAGATCTGA |
|  | A4 | AATTCTAATACGACTCACTATAGGGAGAAGGCTCATTGTTGACCTCTGGGA | CAAGAGCCAATGTTCAGATGGA |
| GII.6 | S2 | AATTCTAATACGACTCACTATAGGGAGAAGGCAGACAAGAGGCCATGTTCA | TCATTGTTGGCCTCTGGTACGA |


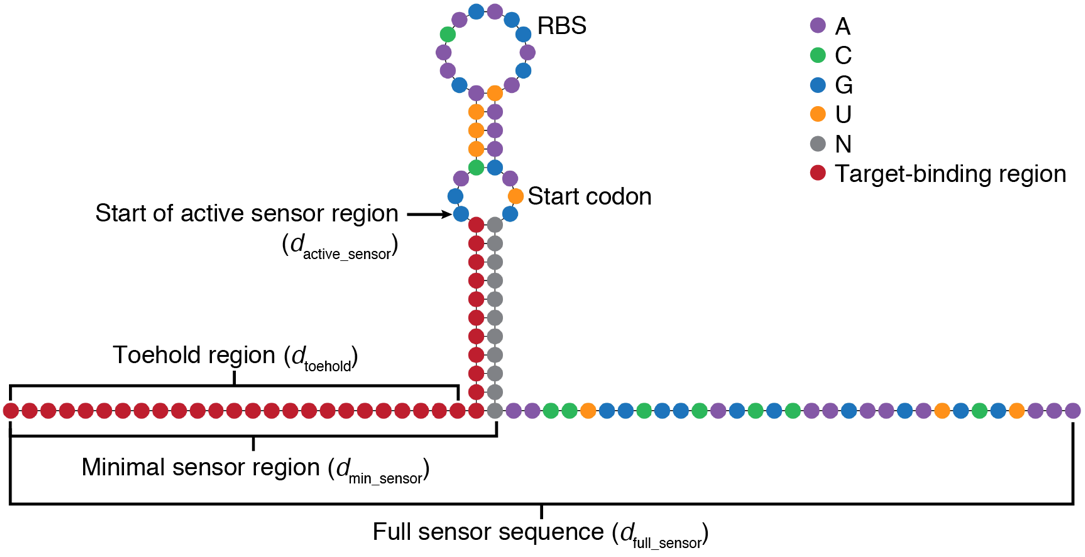


**Supplementary Figure S1.** Schematic of the toehold switch regions used for calculation of design ensemble defect parameters. The full sensor sequence used for calculation of *d*_full_sensor_ is shown along with subsequences spanning the toehold region (*d*_toehold_) and the toehold and hairpin regions (*d*_min_sensor_). The start of the active sensor region is indicated by the arrow and begins at the base that falls 1 nt after the target binding region. The red target-binding region and the gray N bases are the only ones that change for each device.


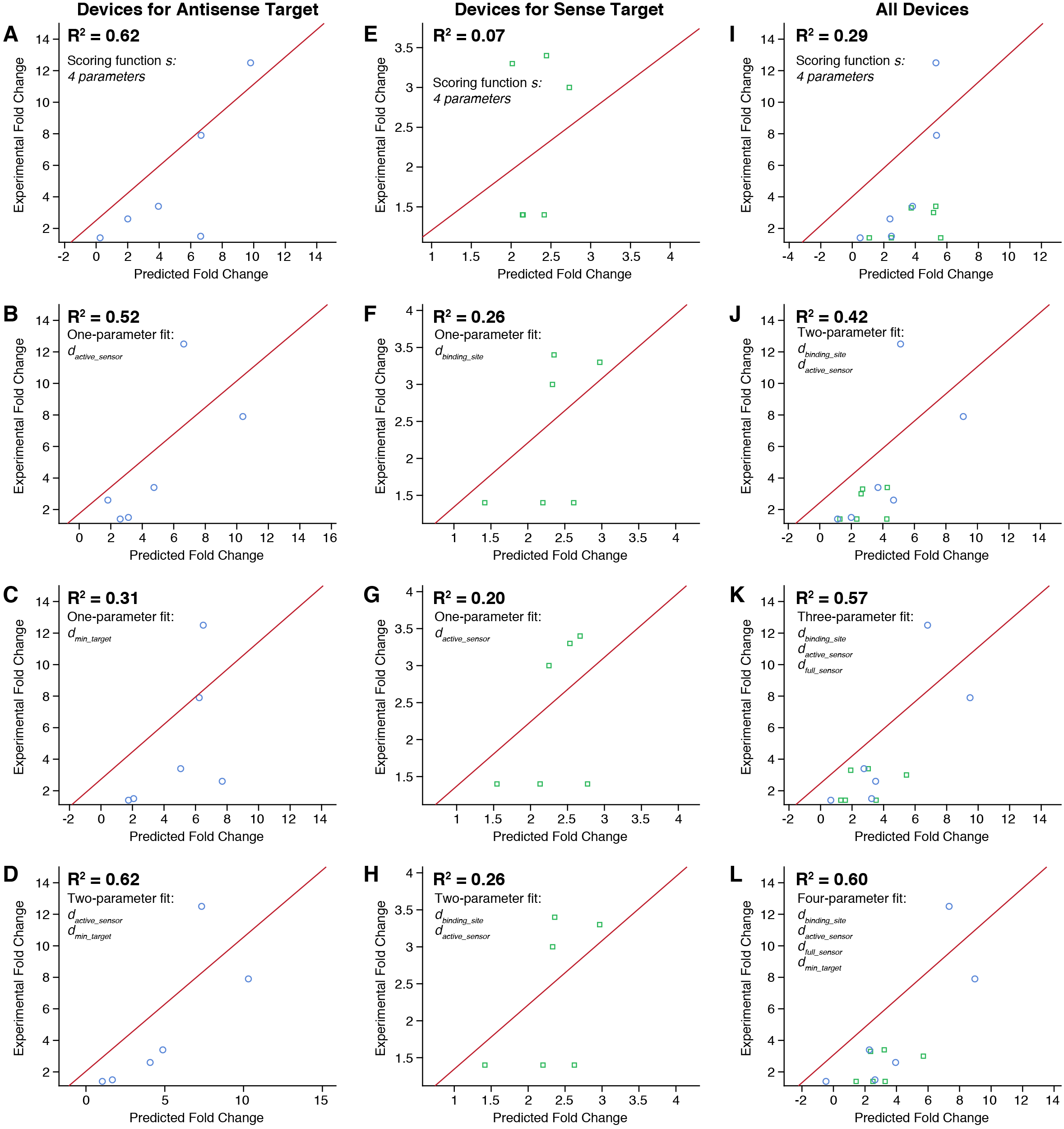


**Supplementary Figure S2.** A series of linear regressions to detect correlations between toehold switch design parameters and their fold change performance for norovirus detection. (A-D) Linear regressions applied only to the set of antisense RNA sensors that provided overall better dynamic range. A two-parameter fit (D) is able to match the correlation with experimental results provided by a four-parameter fit. (E-H) Linear regressions applied only to the set of sense RNA sensors. No correlation is observed with the design scoring function *s* (E). One- and two-parameter fits also do not show much correlation with the experimental results. (I-L) Linear regressions applied to the full set of 12 toehold switches. Use of the same set of predictor variables in the scoring function *s* show limited agreement. However, linear regressions with increasing numbers of parameters provide much stronger correlations (J-L), ultimately reaching R^2^ = 0.60 for a four-parameter regression.


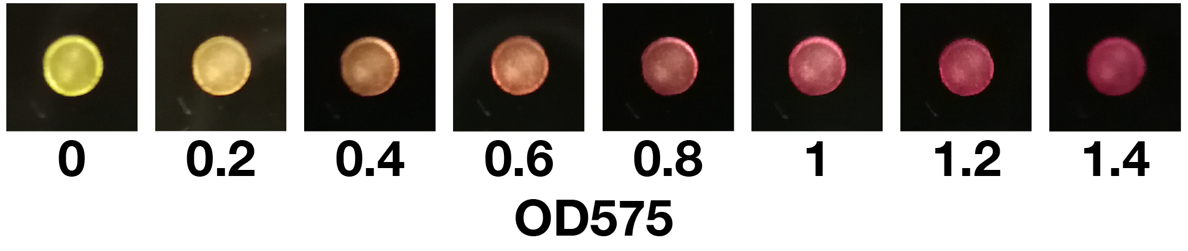


**Supplementary Figure S3.** A series of photographs of paper-based toehold switch reactions using lacZ as the output protein. The colorimetric test results can be easily seen by eye with OD575 down to at least 0.4.


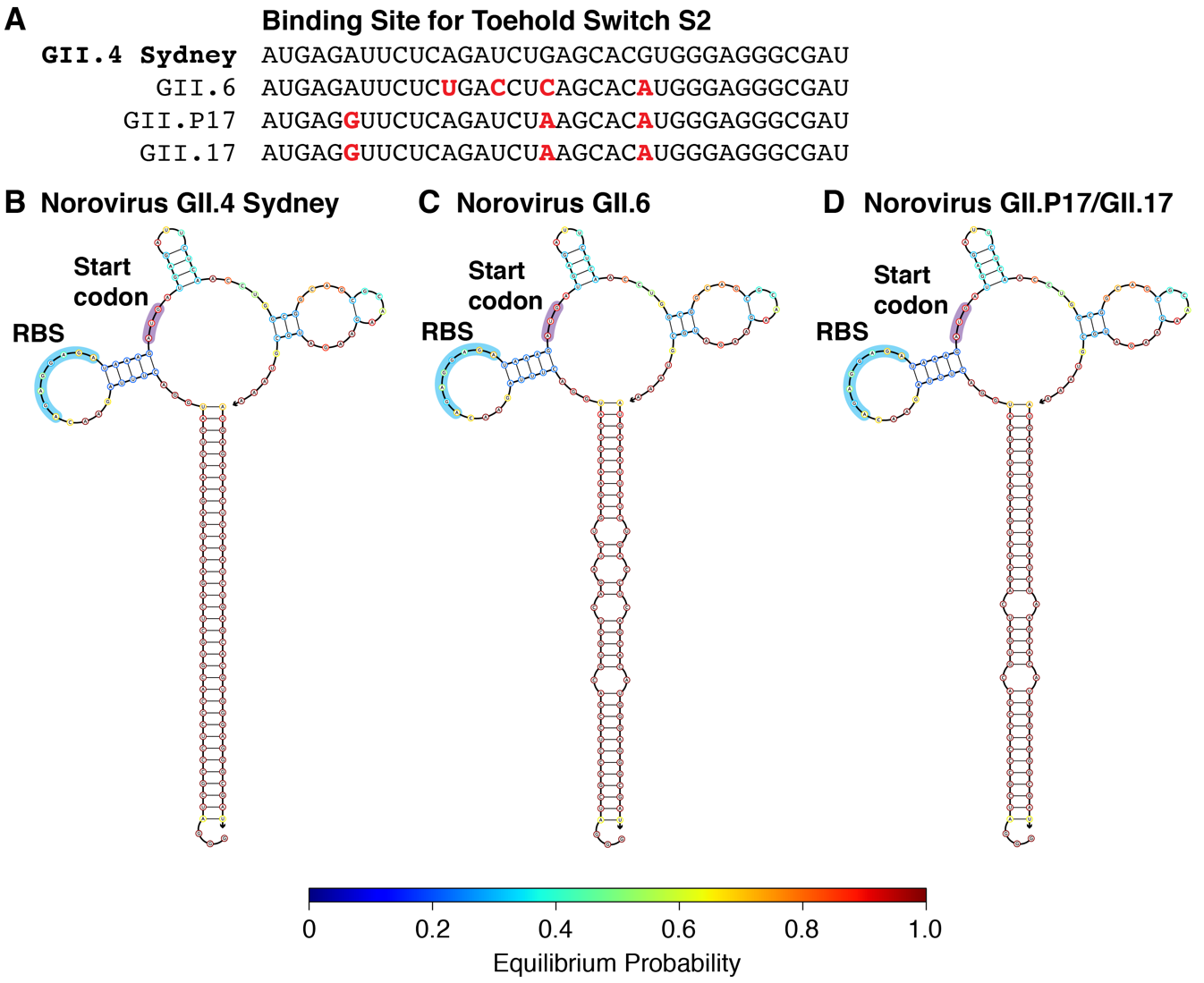


**Supplementary Figure S4.** Comparison of toehold switch interactions with different norovirus GII genotypes. (A) Sequence alignment for strains GII.4 Sydney, GII.6, GII.P17, and GII.17 within the target binding region of the toehold switch. Three or four mutations are present within the 36-nt target domain. Binding sites for GII.P17 and GII.17 are identical. (B-D) Predicted secondary structures of target-switch complexes formed between toehold switch S2 and the three unique target RNAs: GII.4 Sydney (B), GII.6 (C), and GII.P17/GII.17 (D). All targets provide strong binding to the toehold switch. The active sensor region of the switch RNA has the same secondary structure across all three targets, which suggests that translational efficiency will be sufficient to report on target binding.
